# Cystic fibrosis autoantibody signatures associate with *Staphylococcus aureus* lung infection or cystic fibrosis-related diabetes

**DOI:** 10.3389/fimmu.2023.1151422

**Published:** 2023-09-11

**Authors:** Ruchi Yadav, Quan-Zhen Li, Hanwen Huang, S. Louis Bridges, J. Michelle Kahlenberg, Arlene A. Stecenko, Balázs Rada

**Affiliations:** ^1^ Department of Infectious Diseases, College of Veterinary Medicine, The University of Georgia, Athens, GA, United States; ^2^ Department of Immunology and Internal Medicine, University of Texas Southwestern Medical Center, Dallas, TX, United States; ^3^ Department of Epidemiology & Biostatistics, College of Public Health, The University of Georgia, Athens, GA, United States; ^4^ Department of Medicine, Hospital for Special Surgery, Division of Rheumatology, Weill Cornell Medical College, New York, NY, United States; ^5^ Division of Rheumatology, University of Michigan, School of Medicine, Ann Arbor, MI, United States; ^6^ Division of Pulmonology, Asthma, Cystic Fibrosis and Sleep, Department of Pediatrics, Emory University School of Medicine, Atlanta, GA, United States

**Keywords:** CFRD, CF-related diabetes, cystic fibrosis, autoimmunity, autoantibody signature, *Staphylococcus aureus*

## Abstract

**Introduction:**

While cystic fibrosis (CF) lung disease is characterized by persistent inflammation and infections and chronic inflammatory diseases are often accompanied by autoimmunity, autoimmune reactivity in CF has not been studied in depth.

**Methods:**

In this work we undertook an unbiased approach to explore the systemic autoantibody repertoire in CF using autoantibody microarrays.

**Results and discussion:**

Our results show higher levels of several new autoantibodies in the blood of people with CF (PwCF) compared to control subjects. Some of these are IgA autoantibodies targeting neutrophil components or autoantigens linked to neutrophil-mediated tissue damage in CF. We also found that people with CF with higher systemic IgM autoantibody levels have lower prevalence of *S. aureus* infection. On the other hand, IgM autoantibody levels in *S. aureus*-infected PwCF correlate with lung disease severity. Diabetic PwCF have significantly higher levels of IgA autoantibodies in their circulation compared to nondiabetic PwCF and several of their IgM autoantibodies associate with worse lung disease. In contrast, in nondiabetic PwCF blood levels of IgA autoantibodies correlate with lung disease. We have also identified other autoantibodies in CF that associate with *P. aeruginosa* airway infection. In summary, we have identified several new autoantibodies and associations of autoantibody signatures with specific clinical features in CF.

## Introduction

Cystic fibrosis (CF) affects an estimated 160,000 people worldwide (1). CF is a genetic disease that originates from mutations in the Cystic Fibrosis Transmembrane Conductance Regulator (CFTR) gene. CF is a complex, multi-organ disease. While unbelievable progress has been made in the past two decades in understanding CF pathogenesis and in available treatment options, several questions remain unanswered related to the etiologies of organ-specific dysfunction and their potential connection to CF pathophysiology (2).

CF mortality and morbidity are still largely due to lung complications (3–5). In CF, airway disease is characterized by chronic, neutrophil-mediated inflammation, impaired mucociliary clearance and infections by a select group of bacterial pathogens including *Staphylococcus aureus* (*S. aureus*) and *Pseudomonas aeruginosa* (*P. aeruginosa*) (5). While *P. aeruginosa* has been the major respiratory pathogen in CF for years, its prevalence has declined and *S. aureus* infections have become more common in the past ten years (6). *S. aureus* is one of the earliest pathogens discovered in the airways of CF children and remains one of the two main respiratory pathogens persistent throughout the life of people with CF (PwCF). Methicillin-resistant *S. aureus* (MRSA) and small colony variants of *S. aureus* are especially of clinical relevance in CF. MRSA respiratory infection has been associated in CF with more aggressive antibiotic therapy (7), increased hospitalization (8), accelerated decline in lung function (9–11) and increased mortality (12). While *S. aureus* is a very important respiratory pathogen, less is known about its pathogenesis in CF. Beyond a few clinical measures such as the presence of CF-related diabetes (CFRD) or numbers of hospitalizations per year, it is not really known what host factors allow colonization of CF airways by *S. aureus* (6).

In addition to respiratory problems, PwCF also develop a variety of non-pulmonary complications. CFRD, for instance, is a comorbidity occurring in 20% of teen and 50% of adult PwCF (13, 14). CFRD is a distinct form of diabetes and is characterized by a decrease in glucose-regulated insulin secretion, whereas insulin sensitivity is most often normal (7). The development of CFRD is associated with acceleration in decline of lung function and ultimately death due to respiratory failure. Vascular disease, particularly macrovascular disease, is much less common than in other forms of diabetes. The mechanism whereby the development of CFRD markedly worsens CF lung disease is largely unknown (13, 15).

CF lung disease pathogenesis has been classically characterized as the triad of decreased mucociliary clearance, neutrophil-dominated inflammation and polymicrobial infections. Chronic inflammation and infections are also known to increase the chance for the development of autoimmunity in several conditions (16–18). While CF has not been considered traditionally as an autoimmune disease, we thought that the circumstances are present for its potential development in CF. Molecules derived from neutrophils that are chronically present in CF airways could serve as autoantigens. Prior observations reported the presence of anti-neutrophil cytoplasmic antibodies (ANCA) in CF that target cytoplasmic components of neutrophils such as MPO (MPO-ANCA (19, 20)), bacterial permeability increasing protein (BPI-ANCA (21–23)) and proteinase 3 (PR3-ANCA (19, 20)). We have recently identified two new autoantigens in CF: DNA (24) and peptidylarginine deaminase 4 (PAD4) (25, 26). Neutrophil extracellular traps (NETs) represent a likely source of the proteins that potentially could trigger an autoimmune response in CF since all can be found in NETs (27–30). A growing body of literature shows that NETs provide autoantigens in several autoimmune disorders such as rheumatoid arthritis (RA) (31–34), systemic lupus erythematosus (SLE) (35–38), ANCA-related vasculitis (39–42) and autoimmune diabetes (30, 43). Anti-citrullinated protein antibodies are biomarkers in RA pathogenesis (34, 44–46). Nucleosomes trigger autoimmunity in SLE (35). The human blood has natural nuclease activity mediated by DNAse1 (47). Impaired DNAse1-mediated NET degradation has been linked to SLE pathogeneses (48–50) and anti-NET autoantibodies protect NETs from DNAse1 (49). Taken together, these data suggest an underappreciated, potential autoimmune component of CF. Based on all these, direct or indirect, associations we aimed at exploring autoimmunity in CF in detail and hypothesized that novel autoantibodies will be described in PwCF.

Instead of limiting our focus to a few specific autoantibodies, we decided to undertake a more unbiased approach and to map the autoantibody landscape in CF and explore its associations with clinical parameters of the disease. This was done with powerful autoantigen microarrays which assemble a panel of more than 100 autoantigens on a chip. Human sera are exposed to the autoantigens and reveal IgG, IgM, IgA and IgE immunoreactivities. A limited cohort of primarily young adults with CF was tested. Blood samples obtained from non-CF control subjects and from people with well-characterized autoimmune diseases, RA and SLE, were used as comparisons.

Our results revealed several autoantibodies to be elevated in CF compared to non-CF controls. Interestingly, unique autoantibody signatures were found in PwCF that were associated with different clinical phenotypes including *S. aureus* airway infection and CFRD.

## Methods

### Control subjects

All the human subject studies were performed according to the guidelines of the World Medical Association’s Declaration of Helsinki. Control human subjects recruited at the University of Georgia provided informed consent before blood donation according to the protocol UGA# 2012-10769-06. Healthy subjects were chosen to match the sex and age distributions of PwCF and did not carry the diagnosis of CF, RA or SLE based on self-report (**Table 1**; **Supplementary Figure 1A**). Eight to ten milliliters of blood were collected by venipuncture, allowed to clot for thirty minutes and were centrifuged. Serum aliquots were stored frozen until used.

**Table 1:** Sex and age distribution of patient cohorts.

Patient cohort	Cystic Fibrosis (CF)	Healthy controls (HC)	Rheumatoid arthritis (RA)	Systemic Lupus Erythematosus (SLE)
n =	34	26	9	10
Gender				
% female % male	44.1% 55.9%	30.4% 69.6%	60.0% 40.0%	95.2% 4.8%
Age (yrs, mean +/- S.D.)	30.6+/-9.8	29.2+/-11.6	32.5+/-4.8	45.1+/-12.6

### People with cystic fibrosis (PwCF)

CF subjects were recruited from the Children’s + Emory CF Care Center which follows approximately 700 PwCF from the newborn period to old age. PwCF at this Center volunteer to enroll in the CF Biospecimen Repository (CF-BR) (IRB00042577) where biologic samples are collected longitudinally as disease progresses, complications arise, and/or new treatments are initiated. Samples are banked for future studies aimed at understanding CF pathophysiology with the ultimate goal of translating this new knowledge into the design of new therapeutic strategies. For this current study, the biobank was queried for PwCF who had serum samples collected and banked at the time of their clinic appointment at the Care Center and 34 samples were selected randomly that fulfilled all the following inclusion criteria: 1. Diagnosis of CF confirmed by pilocarpine iontophoresis sweat testing and/or CFTR gene mutation analysis showing the presence of two disease causing mutations; 2. Consented for the CF-BR for serum collection; 3. aged 12 years or older at the time of blood draw; 4. Respiratory tract culture done the day of blood collection and results available from the clinical microbiology laboratory; 5. Pulmonary function measured via spirometry on the same day blood was collected; 6. Clinically stable with no acute illnesses within 3 weeks of the clinic visit; 7. On no new medications within 3 weeks of the clinic visit and no new medications, particularly antibiotics, prescribed at the clinic visit; and 8. Sufficient serum volume banked for the autoantibody microarray analysis. Finally, these samples were collected and banked before the highly effective modulator therapy, Trikafta™, was approved for use in CF. The presence or absence of *P. aeruginosa* or *S. aureus* as identified by the clinical microbiology laboratory was noted. The percent predicted forced expiratory volume in one second (FEV_1_%pred) for the day of the clinic visit was recorded as a measure of lung function. The clinical characteristics of PwCF participating in this study are summarized in **Table 2**. Blood was drawn into a silicone coated tube and processed as above until shipped to UGA for analysis.

**Table 2:** Clinical characteristics of CF patients.

Clinical parameter	N (percent)
Lung Function	
FEV_1_ % predicted >80%	18 (53%)
FEV_1_ % predicted 60-80%	8 (24%)
FEV_1_ % predicted 40-60%	7 (20%)
FEV_1_ % predicted <40%	1 (3%)
Sputum or Throat Culture Microbiology	
P. *aeruginosa* positive	22 (65%)
mucoid	13 (38%)
non-mucoid	5 (15%)
mucoid/non-mucoid	2 (6%)
unreported	2 (6%)
*S. aureus* positive	19 (56%)
MRSA	11 (32%)
MSSA	8 (24%)
F508del copy number	
2 F508del alleles	15 (44%)
1 F508del allele	11 (32%)
0 F508del alleles	3 (9%)
undetermined	5 (15%)
CF-related diabetes (CFRD)	15 (44%)
Pancreatic exocrine insufficiency	34 (100%)
Musculoskeletal pain reported	7 (21%)

### SLE patients

SLE patients meeting four or more of the 1997 American College of Rheumatology lupus criteria (51) were recruited from the University of Michigan lupus clinic under IRB-Med 00066116. All patients underwent written, informed consent. Five mL of blood was collected in a serum separator tube and processed as above.

### RA patients

Sera from RA patients were obtained from the University of Alabama at Birmingham (UAB) Rheumatology Arthritis Database and Repository. All patients met the 1987 ARA (now ACR) or 2010 ACR/EULAR classification criteria. All data and samples were obtained in accordance with the UAB Institutional Review Board for Human Use (IRB). Standard techniques for venipuncture and isolation of serum were used as detailed above, and aliquots were shipped on dry ice for analysis.

### Autoantigen microarray

Autoantibody profiling was performed using an existing autoantigen microarray platform developed at the University of Texas Southwestern Medical Center Microarray Core (52). This platform has the capacity to display large number of antigens on a bio-chip and thereby serves as a high-multiplex screening method for the determination of autoantibody specificities (53). In this study, we designed an autoantigen microarray that contains 117 antigens which were selected based on a survey of the literatures to identify autoantigens implicated in various human autoimmune diseases (**Supplemental Table 1**). The protein array chips coated with these autoantigens were used for profiling four isotypes of autoantibodies, including IgG, IgM, IgA and IgE, in human serum samples. Briefly, serum samples were pre-treated with DNAse-I, then diluted at 1:50 (for serum) and hybridized to protein array plates coated with 117 antigens and 8 controls. The array plates were stained with either (I) Cy3-conjugated anti-human IgG (1:2000, Jackson ImmunoResearch Laboratories, PA) and Cy5-conjugated anti-human IgM (1:2000, Jackson ImmunoResearch Laboratories), or (II) FITC-conjugated anti-human IgE (1:500, BioLegend, CA) and Cy5-conjugated anti-human IgA (1:1000, Jackson ImmunoResearch Laboratories). The fluorescent images were acquired with a Genepix 4200 A scanner (Molecular Devices, San Jose, CA) and converted to signal intensity values using GenePix 7.0 software (Molecular Devices). Background was subtracted and signal normalized to internal controls for IgG, IgM, IgE or IgA, respectively. The final value for each autoantibody was expressed as antibody score (Ab-score), which is calculated based on the normalized signal intensity (NSI) and signal-to-noise ratio (SNR) using the formula: Ab-score = log2(NSI∗SNR + 1).

The normalized and unit variance-scaled Ab-score values were used to generate heat maps, organized by unsupervised hierarchical co-clustering using Cluster and Treeview software (http://bonsai.hgc.jp/~mdehoon/software/cluster/software.htm). In heat maps (**Supplementary Figure 1B**), values were centered by rows; each row in the heatmap represents an autoantibody and each column represents a sample. Yellow color represents the signal intensity higher than the mean value and the blue color represents signal intensity lower than the mean value. Grey or black color represents the signal is close or equal to the mean value of the raw.

In parallel with the above-described antigen array analysis, a whole-chip citrullination was also performed to create a corresponding array with the same 117 autoantigens but all modified by citrullination. A protein citrullination kit (Genecopoeia, MD) was used to produce the corresponding citrullinated arrays. Antigens that had been immobilized to the array chip were incubated with a mixture of 5 human recombinant PAD enzymes (PAD1, PAD2, PAD3, PAD4 and PAD6) to produce citrullinated antigens. The quality of citrullination was then confirmed by antibody-based detection of protein citrullination. Autoantibody reactivities against citrullinated autoantigens were measured in the sera the same way as with the non-citrullinated autoantigens described above.

### Statistical analysis

Results between two subject cohorts were analyzed by Mann-Whitney test while data among more than two cohorts were compared by Kruskal-Wallis test. One-way ANOVA and Tukey’s multiple comparisons test are also considered. Correlation between two parameters was evaluated with Spearman’s rank correlation. Data are expressed as mean plus-minus standard deviation. The correlation coefficient (r) and two-sided *p* values were calculated. To study how our conclusion can be influenced by the confounding variables such as FEV_1_%pred, sex and age, we conducted multivariate regression analysis for major observations. Specifically, multivariate logistic regression was used to study how the binary variables (*S. aureus* infections or CFRD) depend on the measured IgA or IgM scores, plus confounding variables FEV_1_%pred, sex and age of PwCF. Statistically significant differences were considered as *, p<0.05; **, p<0.01; ***, p<0.001. Statistical analysis was carried out with GraphPad Prism version 6.07 for Windows software.

## Results

### PwCF do not have significantly higher levels of autoantibodies of a certain immunoglobulin class

**Figure 1:**
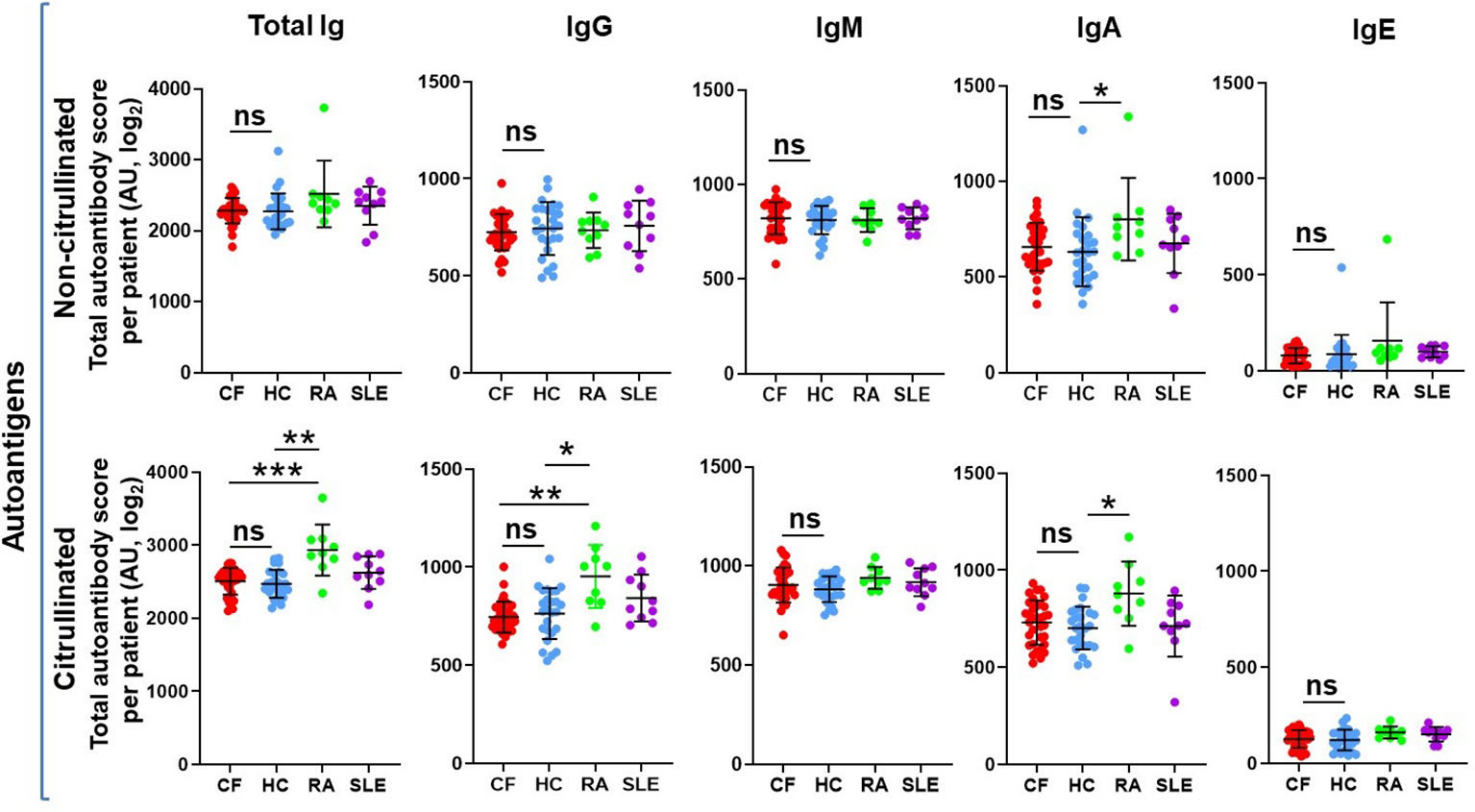
The overall systemic autoantibody landscape in cystic fibrosis. Sera obtained from 34 PwCF, 26 healthy control subjects (HC), 9 RA and 10 SLE patients were subjected to autoantibody microarray analysis. Total autoantibody scores were calculated for each subject, each Ig class and citrullinated or non-citrullinated autoantigens by adding up all the individual fluorescent values of the 117 autoantibodies tested. Each dot represents a separate subject. One-way ANOVA and Kruskal-Wallis test. AU, arbitrary unit; ns, non-significant. *, p<0.05; **, p<0.01; ***, p<0.001.

Autoantibodies directed mainly against neutrophil components have been detected in CF in the past (19–23). Our team identified recently two new autoantigens in CF: PAD4 (25, 26) and DNA (24). Next, to explore the autoimmune landscape in CF in an unbiased way, rather than one autoantibody at a time, we performed autoantibody microarray experiments on blood samples of 34 PwCF (Children’s+Emory CF Care Center), 26 healthy control (HC) subjects (University of Georgia), 9 RA patients (University of Alabama) and 10 SLE patients (University of Michigan) (**Supplementary Figure 1A**). The age and the sex distribution of PwCF corresponded to those of HCs (**Table 1**). Samples were tested for the presence of antibodies against a customized list of 117 autoantigens, several of which are known targets in a variety of autoimmune diseases (**Supplementary Table 1**). The list contained only one foreign, bacterial, antigen as a reference: flagellin Cbir1, a well-known bacterial antigen in Crohn’s disease (54–56). Autoantibodies of the IgA, IgG, IgM and IgE classes were explored and results were received as heatmaps (**Supplementary Figure 1B**). To assess overall autoimmunity, autoantibody scores were calculated for each subject by summing up their fluorescence of each autoantibody signal (**Figure 1**). This was done for each immunoglobulin (Ig) class or for all Ig classes together (Total Ig) (**Figure 1**). We did not observe any significant differences in autoantibody scores of any given Ig class between HC and PwCF (**Figure 1**.). Although relative fluorescence values were measured, they allowed comparison between Ig classes. IgG, IgA and IgM autoimmunity scores were detectable and comparable while the IgE autoantibody scores were negligible (**Figure 1**). There was no correlation observed between total autoantibody levels and age of PwCF (**Supplementary Figure 2**). Thus, PwCF do not have elevated systemic autoantibody levels.

### PwCF do not have elevated levels of anti-citrullinated protein autoantibodies

NETs and PAD4, the enzyme mediating NET formation in neutrophils by generating citrullinated proteins, are abundant in CF airways (26, 57). Anti-citrullinated protein antibodies are characteristic in RA (58). Based on these observations, we next explored whether autoantibodies exist in CF that target citrullinated proteins. The 117 autoantigens were also citrullinated *in vitro* using recombinant PAD4 and exposed to the same human serum samples. The same pattern was true for citrullinated autoantigens as that of non-citrullinated ones (**Figure 1**). As expected, RA sera were characterized by significantly higher levels of IgG, IgA and total autoantibodies against citrullinated autoantigens than HC or PwCF (**Figure 1**) (58, 59). Overall, we conclude that PwCF do not produce autoantibodies against citrullinated autoantigens to a greater degree than HC.

### PwCF have significantly higher levels of specific autoantibodies

**Figure 2:**
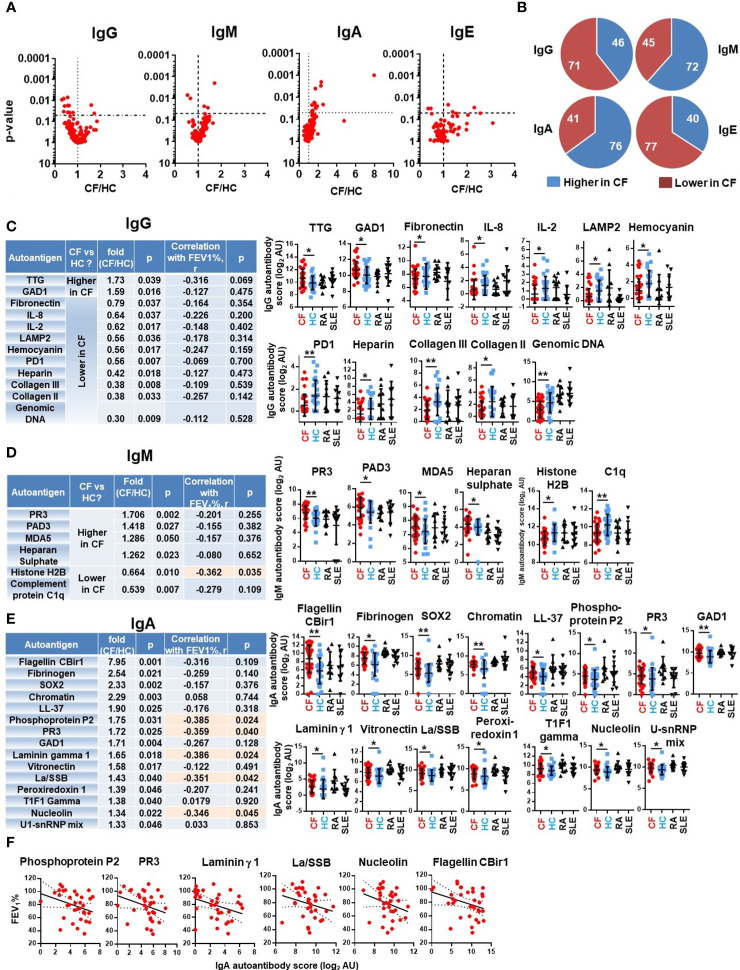
Specific autoantibodies are elevated in CF. **(A)** PwCF and HCs were compared for each autoantibody in each Ig class and the p-value of their difference (Y-axis) was plotted against the ratio of their average values in CF over HC cohorts (CF/HC, X-axis). Each dot represents an autoantibody. Vertical dotted lines indicate a ratio of 1 corresponding to no difference between the two cohorts. Autoantibodies with elevated blood levels in CF compared to HCs are on the right side of this line. Horizontal dotted lines indicate p=0.05, the level of significance. P-values are presented on the Y-axis on a log10-based scale in a reverse order. Autoantibodies with significant differences between CF and HC cohorts are above the horizontal line while non-significant differences are shown below. CF and HC values were compared by Mann-Whitney test. **(B)** The 117 autoantibodies measured for each Ig class were split into two groups: 1) those that are higher in CF (blue), and 2) those that are lower in CF (red), compared to HCs. The average values in the CF cohort were compared to the average values of the HC cohort. **(C)** IgG autoantibodies are listed in the table that have significantly different levels in PwCF compared to HCs and ranked from higher to lower. The CF/HC ratio was calculated based on dividing the average CF value with the average HC value. The first p-value (fourth column from the left) was calculated by Mann-Whitney test for the difference between CF and HC cohorts. Spearman correlation coefficient (r) was calculated between autoantibody levels and lung function (FEV_1_%pred) in the CF cohort for the indicated autoantibodies. The significance of the correlation is indicated in the last column on the right side of the table. The raw fluorescence data are presented for each autoantibody among the four patient cohorts. Each dot represents a separatesubject. **(D)** IgM and **(E)** IgA autoantibodies with significantly different levels between CF and HC cohorts are presented and analyzed the same way as IgG autoantibodies above. Autoantibodies labelled in orange boxes have significant correlation with the severity of CF lung disease. **(F)** IgA autoantibodies with significant negative correlation with CF lung function are shown. Spearman correlation. Each dot represents a separate CF subject. *, p<0.05; **, p<0.01. AU, arbitrary unit; CF, cystic fibrosis; HC, healthy controls; Rheumatoid arthritis; SLE, systemic lupus erythematosus.

No differences were found in the total IgG, IgM, IgA or IgE autoimmune landscape in CF and similarly no such differences were observed in samples of SLE, a well-characterized autoimmune disease (**Figure 1**). We thus then explored whether specific autoantibodies had significantly different levels in PwCF compared to non-CF controls. For each autoantibody, fluorescence values were compared between HCs and PwCF and their CF/HC ratios were plotted against their p-values for each Ig class (**Figure 2A**). Ten IgG autoantibodies were found to be significantly lower in CF than in HCs while two IgG autoantibodies had significantly higher levels in CF (**Figures 2A, C**). In contrast, four IgM autoantibodies had significantly higher, while two IgM autoantibodies had lower levels in CF compared to HC (**Figures 2A, D**). Surprisingly, 14 IgA autoantibodies showed significantly elevated blood levels in CF compared to HCs (**Figures 2A, E**). The same trends were reflected when the number of autoantibodies were counted that were either higher or lower in CF, compared to controls (**Figure 2B**). The fluorescence values of about two-thirds of IgG and IgE autoantibody signals were lower in CF than in HCs (**Figure 2B**). On the contrary, about two-thirds of the IgA and IgM autoantibody fluorescence values were higher in CF compared to the non-CF cohort (**Figure 2B**). The most remarkable difference between PwCF and HCs was demonstrated by IgA autoantibody signals (**Figure 2E**). The largest difference between CF and HC was seen with the anti-flagellin CBir1 signal (**Figure 2E**). This measure served as a reference signal not originating from host but from foreign antigens. A more than 2-fold elevation of IgA autoantibody levels in CF were observed in case of the following autoantigens: fibrinogen, Sex determining region Y-box 2 (SOX2) and chromatin (**Figure 2E**). Neutrophils components (LL-37 and proteinase-3) were also among the top IgA hits (**Figure 2E**). Not only the majority of autoantibody signals higher in CF belonged to the IgA class, but about half of them demonstrated a significant, negative correlation with the lung function of PwCF (FEV_1_%pred) (**Figures 2E, F**). In comparison, only one of the 18 IgG or IgM autoantibodies that had significantly altered levels in CF compared to HCs showed a significant (negative) correlation with lung function (**Figures 2C, D**). These data indicate that PwCF develop an autoimmune response against certain host antigens, the blood levels of some of which interestingly also correlate with the severity of CF lung disease.

### PwCF without S. aureus airway infection have high IgM autoantibody titers

**Figure 3:**
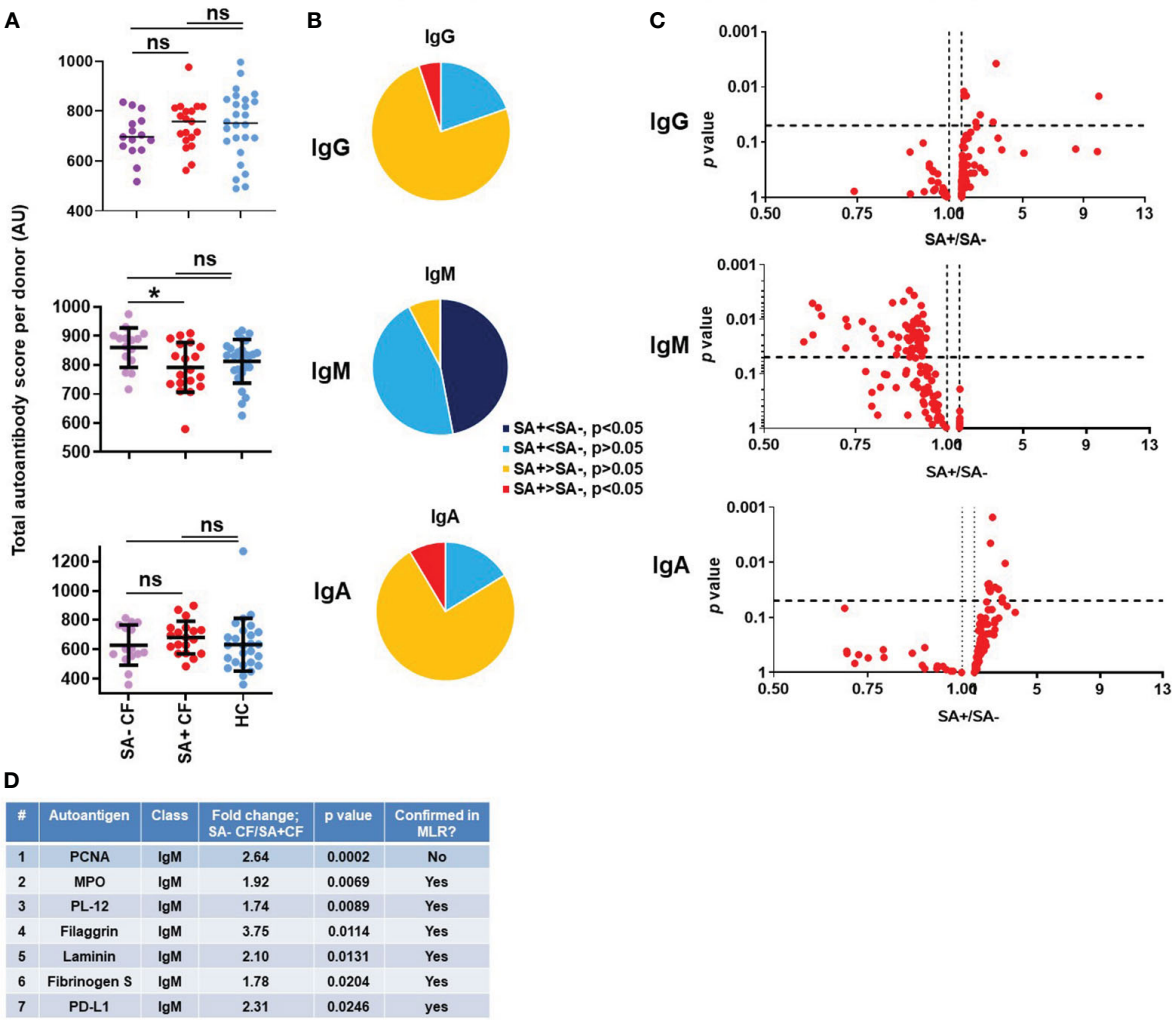
Association of *S. aureus* respiratory infection with a unique IgM autoantibody signature in cystic fibrosis. **(A)** Total IgG, IgM and IgA autoantibody scores were calculated for each subject by summing up the fluorescence values of all 117 autoantibodies and compared between healthy controls (HC, blue), *S. aureus*-negative (SA- CF, purple) and *S. aureus*-positive (SA+ CF, red) CF cohorts. One-way ANOVA, Kruskal-Wallis test. **(B)** The 117 autoantibodies measured for each indicated Ig class were split into four groups based on their average values and the significance of their differences between SA- and SA+ PwCF: 1) significantly lower in SA+ CF (dark blue), 2) non-significantly lower in SA+ CF (light blue), 3) non-significantly higher in SA+ CF (yellow), and 4) significantly higher in SA+ CF (red), compared to SA- PwCF. **(C)** SA- and SA+ PwCF were compared for each autoantibody in each indicated Ig class and the p-value of their difference (Y-axis) was plotted against the ratio of their average values in SA+ CF over SA- CF cohorts (SA+/SA-, X-axis). Each dot represents an autoantibody. Vertical dotted lines indicate a ratio of 1 corresponding to no difference between the two cohorts. Autoantibodies with elevated blood levels in SA+ CF compared to SA- CF are on the right side of this line. Horizontal dotted lines indicate p=0.05, the level of significance. P-values are presented on the Y-axis on a log10-based scale in a reverse order. Autoantibodies with significant differences between SA+ and SA- CF cohorts are above the horizontal line while non-significant differences are shown below. SA+ and SA- CF values were compared by Mann-Whitney test. **(D)** IgM autoantibodies are listed in the table that have significantly different levels in SA+ PwCF compared to SA- PwCF and ranked from higher to lower. The SA-/SA+ ratio was calculated based on dividing the average SA- CF value with the average SA+ CF value for each IgM autoantibody. The p-value was calculated by Mann-Whitney test for the difference between SA- and SA+ CF cohorts. *, p<0.05. MLR, multiple linear regression analysis; ns, non-significant; SA, *Staphylococcus aureus*; CF, cystic fibrosis; HC, healthy control; FEV, forced expiratory volume; AU, arbitrary unit.

*S. aureus* is one of the earliest pathogens recovered in the airways of CF children and, along with *P. aeruginosa*, remains a main respiratory pathogen persistent throughout the life of PwCF (60–62). MRSA, in particular, is associated with accelerated decline in lung function, increased hospitalization and mortality in CF (12, 63). While persistence of MRSA in CF airways has been associated with receiving care at a CF Center, pancreatic insufficiency, CFRD and number of hospitalizations per year (60), no well-defined, molecular host factors are known to date that correlate with *S. aureus* infection (or its absence) in CF. PwCF were considered positive for *S. aureus* in our study if the culture taken at the time of blood draw was positive. In a preliminary study of 99 PwCF, the culture done on the day of the blood draw correctly identified the *S. aureus* status in 80% of the subjects when the status was defined more stringently by the results of at least 4 cultures done in the previous 24 months (Stecenko, unpublished data). Among the PwCF who participated in our study, 56% were positive for *S. aureus* infection, 32% for MRSA and 24% for MSSA airway infection (**Table 2**). As reported in **Figure 1**, the mean IgM autoantibody score per subject, a reflection of the IgM-specific autoimmune response, was not significantly different between CF and HC cohorts. When the CF cohort was split, however, into people with or without *S. aureus* infection, a clear difference was observed between their IgM autoantibody profile: *S. aureus*-negative PwCF (SA- CF) had significantly higher IgM autoantibody scores than *S. aureus*-positive PwCF (SA+ CF) (**Figure 3A**). This difference was again confirmed when comparing the number of IgM autoantibody signals that were lower in PwCF compared to HCs (**Figure 3B**). About half of the IgM autoantibodies are significantly lower in the blood of *S. aureus-*positive PwCF compared to *S. aureus*-negative people (**Figures 3B, C**). **Figure 3C** further confirms this trend in another way when for each autoantibody a ratio was calculated by dividing their values in *S. aureus*-positive PwCF by the one observed in *S. aureus*-negative CF individuals, and this ratio was plotted against the p-value of their difference (**Figure 3C**). This difference in the IgM autoantibody signature was not present in their IgG and IgA autoantibody scores (**Figures 3A–C**). While *S. aureus* typically infects younger PwCF (64), there was no significant difference (p=0.656, Mann-Whitney test) between the age range of *S. aureus*-negative (30.8 ± 10.1 years, mean ± S.D., n=15) and *S. aureus*-positive PwCF (30.4 ± 10.1 years, mean ± S.D., n=19) in our study, so we do not think an age bias drove the observed IgM autoantibody difference among PwCF who are infected, or not, with *S. aureus* (**Supplementary Figure 3A**). Similarly, no difference was observed in the lung function values between the two cohorts of PwCF based on their *S. aureus* status (**Supplementary Figure 3B**). When multivariate logistic regression was also used for the binary variable *S. aureus* infection against total IgM autoantibody score, FEV_1_%pred, sex and age, a p-value of 0.0382 was obtained for total IgM autoantibody score, which means that after controlling for FEV_1_%pred, sex and age, the difference in IgM autoantibody scores between PwCF with or without *S. aureus* respiratory infection still remain significant. Overall, we found a strong association between *S. aureus* infection and low levels of systemic IgM autoantibodies. These results suggest that certain IgM autoantibodies could be protective against *S. aureus* infection in CF and could indicate the first molecular determinants of *S. aureus* lung colonization in the disease.

### The top IgM autoantibodies with higher levels in S. aureus-negative PwCF

The IgM autoantibodies were next ranked according to their *p*-values between the *S. aureus-*positive and *S. aureus-*negative CF cohorts. **Figure 3D** lists the top seven hits. Proliferating cell nuclear antigen (PCNA) stood out amongst all the hits with the strongest association (*p*=0.0002, **Figure 3D**; **Supplementary Figure 4**). Anti-PCNA IgA and IgG autoantibody levels were not affected by *S. aureus* infection status (**Supplementary Figure 4**). When PwCF were split according to their anti-PCNA IgM levels into “low” and “high” groups, low anti-PCNA IgM PwCF were all *S. aureus*-positive while 83% of “high” anti-PCNA IgM PwCF were *S. aureus*-negative. **Figure 3D** shows the detailed results of the top two to seven IgM autoantibodies. It is of note that myeloperoxidase (MPO, #2) is a neutrophil-specific enzyme (65), PD-L1 (# 7) has increased expression in neutrophils in CF airways (66) and hits #4-6 (filaggrin (67), laminin (68), fibrinogen) are components of the airway epithelium, the extracellular matrix or the blood clotting cascade, and are usually linked to neutrophil-mediated tissue injury in pathological conditions. Autoantibodies against PL-12, an alanyl-tRNA-synthetase (69), are present in autoimmune diseases and associate with interstitial lung disease (69, 70). Multivariate logistic regression was next performed for binary variable *S. aureus* infection against FEV_1_%pred, sex, age, CFRD and P. aeruginosa infection for each of the top 7 IgM autoantibodies. While the p-value for anti-PCNA IgM between PwCF with or without *S. aureus* infection became insignificant (p=0.361), it remained significant for the remaining IgM autoantibodies tested after including the indicated, additional variables into the analysis: MPO (p=0.013), PL-12 (p=0.019), filaggrin (p=0.010), laminin (p=0.046), fibrinogen S (p=0.033) and PD-L1 (p=0.018), (**Figure 3D**). Overall, most of the top IgM autoantibodies with higher levels in *S. aureus*-negative PwCF target antigens specific to neutrophils or related to neutrophil-mediated tissue damage.

### The IgM autoantibody score correlates with lung disease in S. aureus-positive PwCF

**Figure 4:**
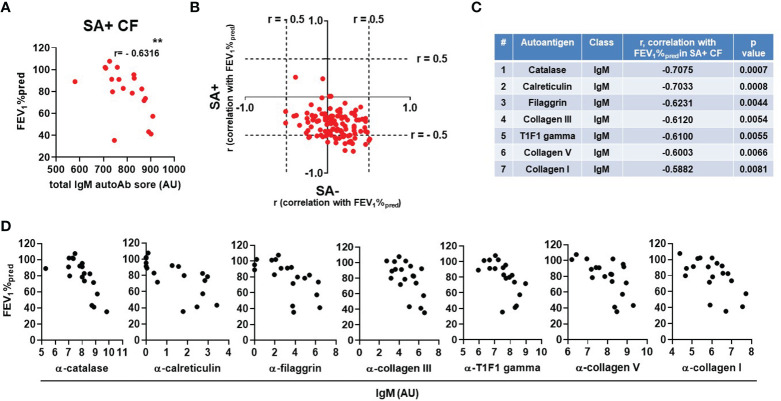
The IgM autoantibody score correlates with lung disease in *S. aureus*-positive PwCF. **(A)** For each SA+ CF subject the total IgM autoantibody score was calculated (X-axis) and correlated with lung function (FEV_1_%pred, Y-axis) (Spearman correlation coefficient). **(B)** For each IgM autoantibody correlation coefficients were calculated with CF lung function (FEV_1_%pred) and plotted against each other in the SA- (X-axis) and SA+ (Y-axis) CF cohorts. Each dot represents an IgM autoantibody. The top 7 IgM autoantibodies are **C)** ranked in the table according to their descending Spearman correlation coefficient values with CF lung function (FEV_1_%pred) in SA+ PwCF and **D)** their correlation data are shown. **, p<0.01. SA, *Staphylococcus aureus*; CF, cystic fibrosis; HC, healthy control; FEV1, forced expiratory volume; AU, arbitrary unit.

We next addressed whether blood levels of IgM autoantibodies show correlation with lung function in CF. In *S. aureus*-negative PwCF, where levels of select IgM autoantibodies are higher, there was no correlation between lung disease and IgM autoantibody levels (**Supplementary Figure 5**). In *S. aureus*-positive PwCF with low total IgM autoantibody titers, however, a surprising negative and strong correlation was observed with lung function (**Figure 4A**). This is further demonstrated when the correlation coefficients between the antibody’s fluorescent signal and lung function are calculated for each IgM autoantibody and plotted against each other between the *S. aureus*-positive and -negative CF patient cohorts (**Figure 4B**). The cloud consisting of red dots is clearly shifted to the right and even more to the bottom indicating a negative correlation between lung disease and several IgM autoantibodies in the *S. aureus*-positive population (**Figure 4B**). **Figure 4C** lists and ranks the top IgM autoantibodies with the strongest negative correlation with lung function in PwCF with *S. aureus* infection. The individual data sets are shown for each of the top seven IgM autoantibodies in **Figure 4D**. Three of these IgM autoantibodies target different forms of collagen (**Figures 4C, D**). In summary, these data indicate a potentially complex role of IgM autoantibodies in CF.

### Higher IgA autoantibody levels characterize CF-related diabetes

**Figure 5:**
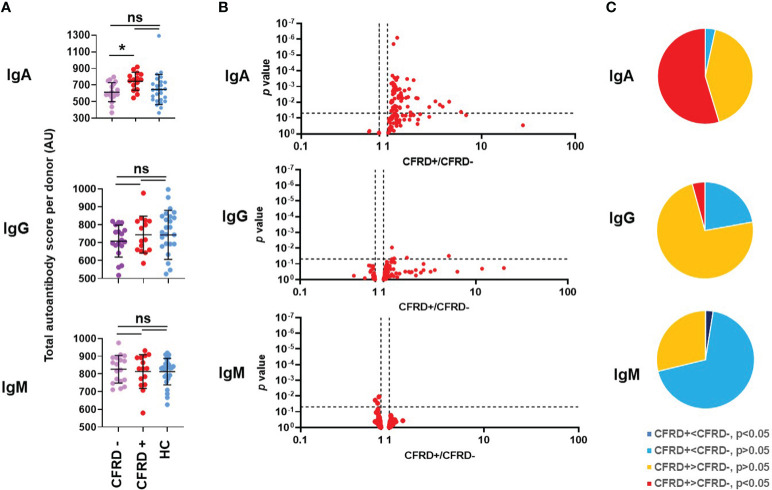
Cystic fibrosis-related diabetes is associated with an elevated systemic IgA autoantibody profile. **(A)** Total IgA, IgG and IgM autoantibody scores were calculated for each subject by summing up the fluorescence values of all 117 autoantibodies and compared between healthy controls (HC, blue), diabetic PwCF (CFRD+, red) and nondiabetic PwCF (CFRD-, purple). Each dot represents a human subject. One-way ANOVA, Kruskal-Wallis test. **(B)** CFRD- and CFRD+ PwCF were compared for each autoantibody in each indicated Ig class and the p-value of their difference (Y-axis) was plotted against the ratio of their average values in CFRD+ CF over CFRD- CF cohorts (CFRD+/CFRD-, X-axis). Each dot represents an autoantibody. Vertical dotted lines indicate a ratio of 1 corresponding to no difference between the two cohorts. Autoantibodies with elevated blood levels in CFRD+ compared to CFRD- are on the right side of this line. Horizontal dotted lines indicate p=0.05, the level of significance. P-values are presented on the Y-axis on a log10-based scale in a reverse order. Autoantibodies with significant differences between CFRD+ and CFRD- CF cohorts are above the horizontal line while non-significant differences are shown below. CFRD+ and CFRD- CF values were compared by Mann-Whitney test. **(C)** The 117 autoantibodies measured for each indicated Ig class were split into four groups based on their average values and the significance of their differences between CFRD- and CFRD+ PwCF: 1) significantly lower in CFRD+ CF (dark blue), 2) non-significantly lower in CFRD+ CF (light blue), 3) non-significantly higher in CFRD+ CF (yellow), and 4) significantly higher in CFRD+ CF (red), compared to CFRD- PwCF. *, p<0.05. Ns, non-significant; CF, cystic fibrosis; CFRD, CF-related diabetes; HC, healthy control; AU, arbitrary unit.

CFRD represents a unique form of diabetes and it is unclear why so many people with CF develop diabetes (7–9). We wished to address a potential role of autoimmunity in CFRD given the association between autoimmunity and other forms of diabetes (71). CFRD was diagnosed by a CF endocrinologist using CF Foundation guidelines which require a combination of two positive tests (oral glucose tolerance test, OGTT, with the fasting glucose greater than 125 mg/dl and/or the two-hour value greater than 199 mg/dl; hemoglobin A1c greater than 6.1%; and/or random plasma glucose of 200 mg/dl or higher) done when the person is clinically stable. CF without diabetes was defined as the absence of the diagnosis of CFRD by the CF endocrinologist rather than by OGTT criteria as during the time the blood samples were collected, the rate of performing annual OGTTs to screen for CFRD in our Center was less than 50% of eligible people. However, the fact that CFRD was diagnosed in 44% of the CF cohort (**Table 2**) suggests that CFRD was not significantly underdiagnosed in our study. Based on these definitions, the CF cohort in our study was split into two groups for analysis: PwCF with CFRD (CFRD +) and those without (CFRD -). When the total autoantibody scores of each Ig class were analyzed, a significant difference could only be observed for IgA autoantibodies between the diabetic and nondiabetic CF cohorts (**Figure 5A**). This was not the case for IgG and IgM autoantibody scores (**Figure 5A**). There were only a few IgG and IgM autoantibody signals that were significantly different between diabetic and nondiabetic PwCF (**Figure 5B**). However, when IgA autoantibody levels were analyzed, a strong association with CFRD was uncovered (**Figure 5B**). A large portion of the IgA autoantibodies had higher levels in diabetic PwCF than in CF without diabetes and more than half of them were significantly elevated (**Figure 5C**). **Supplementary Figure 6A** presents the raw data for each IgA autoantibody that showed a significant difference between CFRD- and CFRD+. In contrast, only three IgG, two IgM and nine IgE autoantibody signals were dependent on the diabetic status of PwCF (**Supplementary Figure 6B**). CFRD typically manifests at older age (72). There was no significant difference (p=0.056, Mann-Whitney test) between the age range of CFRD-negative (27.7 ± 9.7 years, mean ± S.D., n=19) and CFRD-positive PwCF (34.2 ± 9.1 years, mean ± S.D., n=15) in our cohort, although the p-value was close to the level of significance. Multivariate logistic regression analysis was next undertaken for the binary variable CFRD against the total IgA autoantibody score, FEV_1_%pred, sex and age. A p-value of 0.0069 for the total IgA autoantibody score was recovered confirming that the difference remains significant after controlling for FEV_1_%pred, sex and age of PwCF. Overall, a novel association between systemic IgA autoimmunity and CFRD has been proposed in our study.

### Autoantibodies linked to P. aeruginosa airway infection in CF

In addition to *S. aureus*, *P. aeruginosa* represents another major respiratory pathogen in CF (73). IgA autoantibodies targeting the bacterial permeability increasing protein (BPI) have been found to be associated with *P. aeruginosa* infection in CF (74). We confirmed this in our microarray data as anti-BPI IgA is one of our hits too (**Supplementary Figure 7A**). An additional, five IgA and five IgG autoantibodies could be identified in our scan that were all higher in PwCF infected with *P. aeruginosa* compared to *P. aeruginosa*-free CF individuals (**Supplementary Figure 7A**). We were also curious to see whether any autoantibodies associate with the mucoid status of *P. aeruginosa*. Only *P. aeruginosa*-positive PwCF were analyzed here by grouping them into those infected with mucoid versus non-mucoid *P. aeruginosa*. Interestingly, we could identify four IgG autoantibodies that had significantly different scores between the two cohorts (**Supplementary Figure 7B**). Three of them (CTLA-4, ferritin and muscarinic receptor) were higher in PwCF with non-mucoid *P. aeruginosa* while anti-Nup62 IgG scores were higher with mucoid *P. aeruginosa*.

Overall, these results reveal several novel associations between specific autoantibodies and different clinical phenotypes in PwCF.

## Discussion

CF is not considered an autoimmune disease. Sporadic observations detected the presence of antibodies targeting host proteins in CF over the past decades including BPI (21–23), MPO (19, 20), proteinase 3 (10, 11), PAD4 (25, 26) and DNA (24). These autoantigens are either proteins highly and/or specifically expressed in neutrophils or extracellular DNA whose major source in CF airways has been thought to be neutrophils undergoing NET formation (75–78). Thus, neutrophils have been the prime suspect as the source of these autoantigens in CF (79, 80). Antineutrophil cytoplasmic antibodies (ANCA) have been described early and linked to several diseases including CF (81–84). Among many potential mechanisms exposing autoantigens from neutrophils to the immune system, NET formation has been proposed as a likely route as it delivers all antigens (proteins and DNA) reported in CF to the extracellular space (27, 79, 81). NETs were shown to provide autoantigens in several autoimmune disorders such as RA (31–34), SLE (35–38), ANCA-related vasculitis (39–42) and autoimmune diabetes (30, 43). We have recently reported the presence of autoantibodies against peptidyl arginine deiminase type IV (PAD4), an enzyme highly expressed in neutrophils that is essential for histone citrullination and subsequent NET release (25, 26), and against human DNA (24).

While a handful of autoantibodies have been reported in CF in the past, it is not clear that they have any role in the disease as even healthy people have elevated levels of some autoantibodies without any clear health benefit or damage (85). Autoimmunity does not necessarily mean autoimmune disease (85). Therefore, the next important step is to prove that these autoantibodies do have a role in pathogenesis, or on the contrary, have beneficial effects. Such an indication can be obtained from data correlating autoantibody levels with clinical parameters of the disease. While correlation does not mean causality, it is a good first step in exploring the potential clinical importance of an autoantibody. In CF, for instance, several of the autoantibodies reported so far have been correlated with worsening lung disease (24, 25, 79) or *P. aeruginosa* infection (25, 80). A surprising, beneficial role has been proposed for one BPI-ANCA autoantibody via promoting *P. aeruginosa* phagocytosis by neutrophils in a CD18-dependent manner (86, 87). No other studies have suggested a beneficial or pathogenic role of autoantibodies in CF, especially in *in vivo* experimental systems.

Here we employed an unbiased approach to study the autoantibody landscape in CF using autoantigen microarrays that have already been successfully used by us in the research of autoimmune diseases (52, 53, 88) and chronic inflammatory lung diseases (89, 90). We did not find elevated total blood levels of autoantibodies of a certain Ig class in CF. Nor did we observe that citrullinated proteins represent autoantigens in CF as is the case in other diseases such as RA (58). This latter finding is in line with prior reports by us (25) and others (79) when no difference in total ACPA levels were seen between PwCF and control individuals as measured by ELISA. At the same time, these data are somewhat surprising since neutrophils, NETs and PAD4 are all present in CF airways (26, 78) and autoantibodies targeting DNA and PAD4 are also found in CF (24–26). These results led us to determine if specific autoantibodies were elevated in CF and if so, were they associated with a particular clinical phenotype. The following paragraphs discuss the potential significance of this avenue of investigation.

We identified two IgG, four IgM and 14 IgA autoantibodies that have elevated blood levels in PwCF compared to control individuals. Some of the targets of these autoantibodies have already been known in CF: anti-chromatin IgA (including DNA) (24) and anti-proteinase 3 IgA and IgM (82). Interestingly, while BPI-ANCA antibodies have been reported in CF (21, 23) and BPI was included onto the microarray, we failed to detect significantly higher anti-BPI levels in CF in case of any Ig class. Similarly, MPO-ANCAs have been reported in CF (19, 20), but our study did not detect anti-MPO autoantibodies to be elevated in PwCF compared to HCs, but did in *S. aureus*-negative PwCF compared to *S. aureus*-infected PwCF (anti-MPO IgM). This could be because some prior studies failed to detect or detected only low levels of MPO-ANCAs in a small portion of their CF cohorts (21, 82). Our data showing lower anti-MPO IgM levels in PwCF infected with *S. aureus* could also indicate that autoimmunity to MPO might decrease with the higher proportion of PwCF being positive for *S. aureus* nowadays compared to years or decades ago. IgG autoantibodies to tissue transglutaminase (TTG), the autoantigen of coeliac disease (91), were found to be higher in CF blood than HC. NETs have been described to induce the production of autoantigens by airway epithelial cells including TTG (92) and thus could contribute to TTG release in CF to induce autoimmunity. Type 1 or insulin-dependent diabetes mellitus (T1DM, IDDM) is an autoimmune disease (93). The four main autoantibodies detected in people with T1DM are those targeting GAD65, tyrosyl phosphatase (IA-2), insulin (IAA) and zinc transporter (ZnT8) (94). Our results indicate that IgG and IgA autoantibodies targeting glutamic acid decarboxylase 1 (GAD1 or GAD67), a protein closely related to GAD65, are significantly elevated in CF, and anti-GAD1 IgA autoantibodies are also significantly associated with CFRD. While GAD1 autoantibodies have been detected in people with T1DM (95), GAD65 remains the main autoantigen, not GAD1, in T1DM (96). We also found that IgM autoantibodies targeting peptidylarginine deiminase 3 (PAD3), melanoma differentiation-associated protein 5 (MDA5) and heparan sulphate are elevated in CF. Anti-PAD3 antibodies have been found in a subset of RA patients and associated with higher disease activity and joint damage scores (97). MDA5 functions as a pattern recognition receptor detecting long dsRNA and anti-MDA5 autoantibodies have been associated with dermatomyositis or polymyositis patients (98, 99). The most autoantibodies elevated in CF belonged to the IgA class. The antimicrobial anti-flagellin CBir1 IgA antibody was significantly elevated in CF indicating a strong general immune response to bacterial flagellin, most likely stemming from *P. aeruginosa* (100), as indicated previously for anti-*P. aeruginosa* flagellin IgG antibodies (101). Autoantibodies against fibrinogen have been implicated in the pathogenesis of inflammatory arthritis in a mouse model (102). Sex determining region Y-box 2 (SOX2) is a transcription factor important in the maintenance of self-renewal, or pluripotency, of undifferentiated embryonic stem cells and SOX2 autoantibodies are being investigated -together with several others- as early detection signals for different cancers (103). Phosphoprotein P2 is one of the three acidic phosphorylated proteins found in the eukaryotic ribosomes (104) and autoantibodies targeting them have been detected in SLE and associated with nephritis (105). LL-37 is an antimicrobial peptide mainly secreted by neutrophils and airway epithelial cells and promote antibacterial innate immunity at mucosal surfaces including the conducting airways (106, 107). LL-37 binds to NETs and anti-LL-37 autoantibodies have been detected in SLE (108). Laminin gamma 1 is an extracellular matrix glycoprotein and main component of epithelial basement membranes besides collagens including the dermal-epidermal junction. Anti-laminin-γ1 (lam-γ1) pemphigoid, an immunobullous disorder is characterized by anti-laminin G1 IgG antibodies (109). Vitronectin is another component of the extracellular matrix that has been shown to bind to *P. aeruginosa* CF isolates and enhance their adhesion to host epithelial cells (110). La/SSB is Lupus La protein also called Sjögren’s syndrome type B antigen (SS-B), is involved in different aspects of RNA metabolism and its reactivity with autoantibodies is characteristic in patients with systemic autoimmune diseases, SLE and Sjögren’s syndrome (111, 112). La/SSB binds to nucleolin whose main localization is the nucleolus but is unconventionally secreted and serves as a receptor or c-receptor for various bacteria and viruses (113). Anti-nucleolin autoantibodies have been detected in a variety of autoimmune conditions (114). Peroxiredoxin 1 is a redox enzyme that belongs to the family of peroxiredoxins with diverse roles in protection against oxidative stress, redox signaling, protein folding etc. (115, 116). Its role in CF or autoimmunity remains to be determined. Anti-transcription intermediary factor 1 (TIF1)-γ autoantibodies have been strongly associated with cancer-associated dermatomyositis (117, 118). The U1 small nuclear ribonucleoprotein particle (snRNP) complex is a target in SLE and mixed connective tissue disease (119). These hits indicate that neutrophil components or molecules exposed due to neutrophil-mediated tissue damage provide new autoantigens in CF at mucosal sites, likely at the respiratory mucosa. In addition, blood levels of five of these IgA autoantibodies correlate with CF lung disease that further indicates their potential contribution to CF lung disease progression.

Our results indicate a strong association between high IgM autoantibody levels and absence of *S. aureus* infection in PwCF. Neutrophils represent the most important immune cell type fighting *S. aureus* (120–122). Antibody- or complement-enhanced phagocytosis and the associated respiratory burst represent the main mechanism by which neutrophils kill *S. aureus* (120, 121, 123). Neutrophil-mediated killing of *S. aureus* in CF is impaired (60–62). The reason for this remains unclear. *S. aureus* has been detected inside neutrophils in CF airways indicating that phagocytosis occurs to some extent (124).

IgM antibodies are the most potent Ig class activating the complement system (125). The complement system is not capable of lysing *S. aureus*, a Gram positive organism with a thick cell wall, but acts as a strong opsonin for phagocytes (120). IgM autoantibodies could bind to *S. aureus* to increase its neutralization, complement-mediated opsonization and subsequent phagocytic killing (126). IgM autoantibodies (made by B-2 cells in a T cell-dependent fashion) could bind to their specific autoantigen target on the surface of *S. aureus* since fibrinogen is bound to the surface of *S. aureus* (127) and components of lysed neutrophils released into the airway lumen could also be attached to *S. aureus* (MPO, PCNA, PD-L1). *S. aureus* binds host laminins to promote its binding to epithelial surfaces (128). A clinical link between filaggrin mutations and *S. aureus* infection in the skin has been established (129). Anti-PD-L1 antibodies were shown to protect mice against infection with common bacterial pathogens after burn injury including *S. aureus* (130). Natural IgM autoantibodies (made by B-1 cells in a T cell-independent fashion) could bind *S. aureus* nonspecifically since they are polyreactive with low affinity but high avidity (pentamers) and have been shown to bind microbial molecules (131, 132). IgM autoantibodies and subsequent C1q deposition could also label *S. aureus*-phagocytosing, apoptotic neutrophils to increase their apoptotic clearance by macrophages (both IgM- and C1q-deficient mice have increased apoptotic cells and develop SLE-like autoimmune disease (133, 134)). Delayed apoptosis of CF neutrophils is well-documented (134–136). Overall, several mechanisms could lead to the observed association between *S. aureus* infection and IgM autoimmunity in CF.

In *S. aureus*-infected PwCF with low IgM autoantibody titers, IgM autoantibodies targeting seven different antigens were found to be related to lung disease. Collagen I, III and V are components of the extracellular matrix and could be exposed due to neutrophil-mediated tissue damage in CF airways. Filaggrin is a protein that binds keratin fibers in epithelial cells and filaggrin mutations have been associated with predisposition to eczema, a disorder of the skin where *S. aureus* is a standard commensal or pathogen (137). Filaggrin was reported to reduce *S. aureus* uptake by skin epithelial cells (138). Anti-filaggrin IgM autoantibodies could enhance *S. aureus* uptake in airway epithelial cells. Calreticulin resides in the lumen of the endoplasmic reticulum and performs two main functions, Ca^2+^ binding and protein chaperoning, a function clearly impaired in CF, a protein misfolding disease (139). Anti-calreticulin autoantibodies were reported in a cohort of idiopathic inflammatory myopathy patients and associated with malignancy (140). IgM autoantibodies targeting catalase could diminish the antioxidant potential in the airways and promote oxidative stress-mediated lung disease in CF (141).

The exact etiology of CFRD remains incompletely understood despite several theories (13). CFRD is not considered an autoimmune disease despite occasional studies supporting this view (13). Immune cells autoreactive to GAD65, a known beta-cell autoantigen in type 1 diabetes, were found in people with CFRD (142). The reports on the frequencies of beta-cell autoantibodies in CFRD compared to type 1 diabetes are controversial (143, 144). In our work, CFRD is characterized by a general enhancement of IgA autoantibodies compared to nondiabetic patients in the same CF cohort. Whether this correlation represents a causative relationship between CFRD and autoimmunity, remains to be determined in the future. IgA autoantibodies produced in the lung mucosa could bind to targets in the pancreas to lead to the development of CFRD. IgA autoantibodies could also be generated by long-lived plasmablasts in the bone marrow and released in the circulation to result in the same effect. On the other hand, the primary development of CFRD could lead to an IgA-biased autoimmunity in CF.

There are several limitations to this study. This work was done on a single CF cohort of a limited size. This study will have to be repeated on independent CF cohorts and on larger number of subjects to explore whether the interesting observations made here are reproducible. This study only included samples obtained primarily from young adults with CF. To expand on these observations, autoimmunity in younger PwCF will have to be explored to learn more about the development of CF autoimmunity. Another limitation is that while the array included 117 autoantigens, which is a relatively large number, it still represents a sample of the overall possible several thousand potential autoantigen targets. Therefore, results could also be influenced by the list of autoantigens printed on the array.

Overall, our study delivered several novel observations related to CF autoimmunity that have the potential to lead to a better understanding of CF disease pathogenesis and to potentially lay down the foundation for future novel therapeutic approaches for PwCF.

## Data availability statement

The datasets generated and analyzed during the current study are available in the Dryad online repository: https://doi.org/10.5061/dryad.3bk3j9kr1.

## Ethics statement

The studies involving humans were approved by Institutional Review Boards of University of Georgia and Emory University. The studies were conducted in accordance with the local legislation and institutional requirements. The participants provided their written informed consent to participate in this study.

## Author contributions

Project conceptualization and coordination: BR. Providing clinical samples: AS, JK, SB and BR. Sample preparation: RY. Microarray hybridization: Q-ZL. Data analysis: BR, RY, HH. Manuscript writing: B.R. Manuscript revision: RY, Q-ZL, SB, JK, AS, BR. All authors contributed to the article and approved the submitted version.
