# Supplementary material for: Cystic fibrosis autoantibody signatures associate with Staphylococcus aureus lung infection or cystic fibrosis-related diabetes
Source: Front Immunol. 2023 Sep 11;14:1151422. doi: 10.3389/fimmu.2023.1151422 (PMC10519797; doi:10.3389/fimmu.2023.1151422)
Supplement: Supplementary file 1 [file DataSheet_1.pdf]

Supplementary material

Supplementary table 1.

Customized list of autoantigens

#	Autoantigen ID	#	Autoantigen ID	#	Autoantigen ID
1	ANXA1	44	Genomic DNA	87	PAD2
2	ANXA2	45	Hemocyanin	88	PAD3
3	APEX1	46	Heparan Sulphate	89	PAD4
4	Apolipoprotein B/E	47	Heparin	90	PCNA
5	Azurocidin	48	Histone-total	91	PD-L1
6	beta-glucuronidase	49	Histone H1	92	PD1
7	beta2-microglobulin	50	Histone H2A	93	peroxiredoxin 1
8	beta4 integrin	51	Histone H2B	94	Phophatidylinositol
9	BPI	52	Histone H3	95	Phosphoprotein P0
10	Calreticulin	53	Histone H4	96	Phosphoprotein P1
11	Cardiolipin	54	HMGB1	97	Phosphoprotein P2
12	Catalase	55	HMGB2	98	PL-12
13	Cathepsin G	56	Hsp70	99	PL-7
14	CCL11	57	HSPG	100	PM/Scl-75
15	CCL2	58	IFN-a2	101	PR3
16	CCL3	59	IFN-r	102	Ro/SSA (52+60)
17	CENP-A	60	IL-12 (p70)	103	Salivary Gland Protein 1 (SP1)
18	CENP-B	61	IL-17A	104	Sm/RNP
19	Chromatin	62	IL-1a	105	SmD
20	Collagen I	63	IL-1b	106	SOX2
21	Collagen II	64	IL-2	107	SRP54
22	Collagen III	65	IL-6	108	ssDNA
23	Collagen IV	66	IL-8	109	T1F1 Gamma
24	Collagen V	67	Insulin	110	Thyroglobulin
25	Collagen VI	68	Jo-1	111	TNF-a
26	Complement protein C1q	69	KU(P70/P80)	112	Topoisomerase I
27	CTLA4	70	La/SSB	113	TPO
28	CXCL10	71	lactoferrin	114	TTG
29	Cytokeratin 19 Ag	72	Laminin	115	U1-snRNP mix
30	dsDNA	73	Laminin gamma 1	116	Vimentin
31	EGF Receptor	74	LAMP2	117	Vitronectin
32	Elastase	75	LL-37		
33	Enolase	76	MAGEA4		
34	Ferritin	77	MDA5		
35	Fibrilliarin	78	Mi-2		
36	Fibrinogen	79	MMP-9		
37	Fibrinogen IV	80	Muscarinic receptor		
38	Fibrinogen S	81	Myeloperoxidase		
39	Fibronectin	82	Myosin		
40	Filaggrin	83	Nucleolin		
41	Flagellin CBir1	84	Nucleosome		
42	GAD1	85	Nup 62		
43	Galectin	86	PAD1		

Supplementary figure 1. Fluorescent heatmaps of the autoantibody microarrays analyzed in this study.

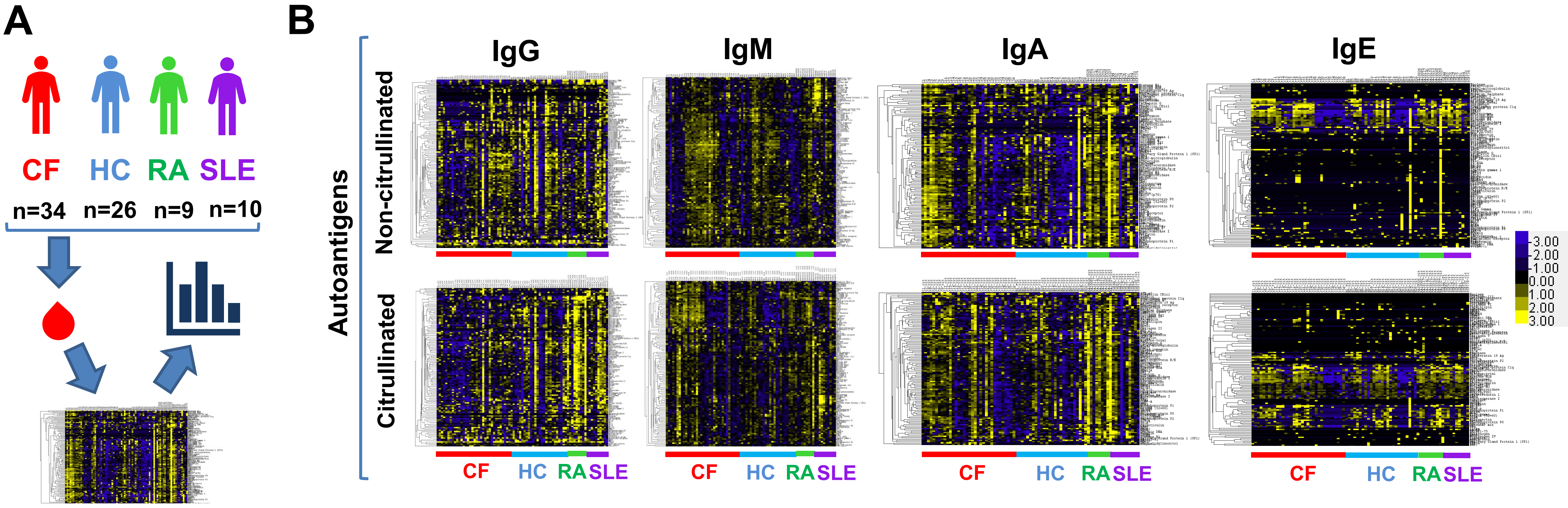

A) Scheme of the recruited patient cohorts (CF, cystic fibrosis; HC, healthy controls; Rheumatoid arthritis; SLE, systemic lupus erythematosus) and the overall sample process. **B)** Fluorescent heatmaps of the eight autoantibody microarrays are shown. 117 custom-selected autoantigens were spotted on chips, left nonmodified or citrullinated in vitro and exposed to human sera from the indicated patient cohorts. Bound IgG, IgM, IgA and IgE autoantibody signals were developed with Ig class-specific secondary antibodies labelled with specific fluorochromes.

Supplementary figure 2. Total autoantibody scores do not correlate with age in young adult PwCF.

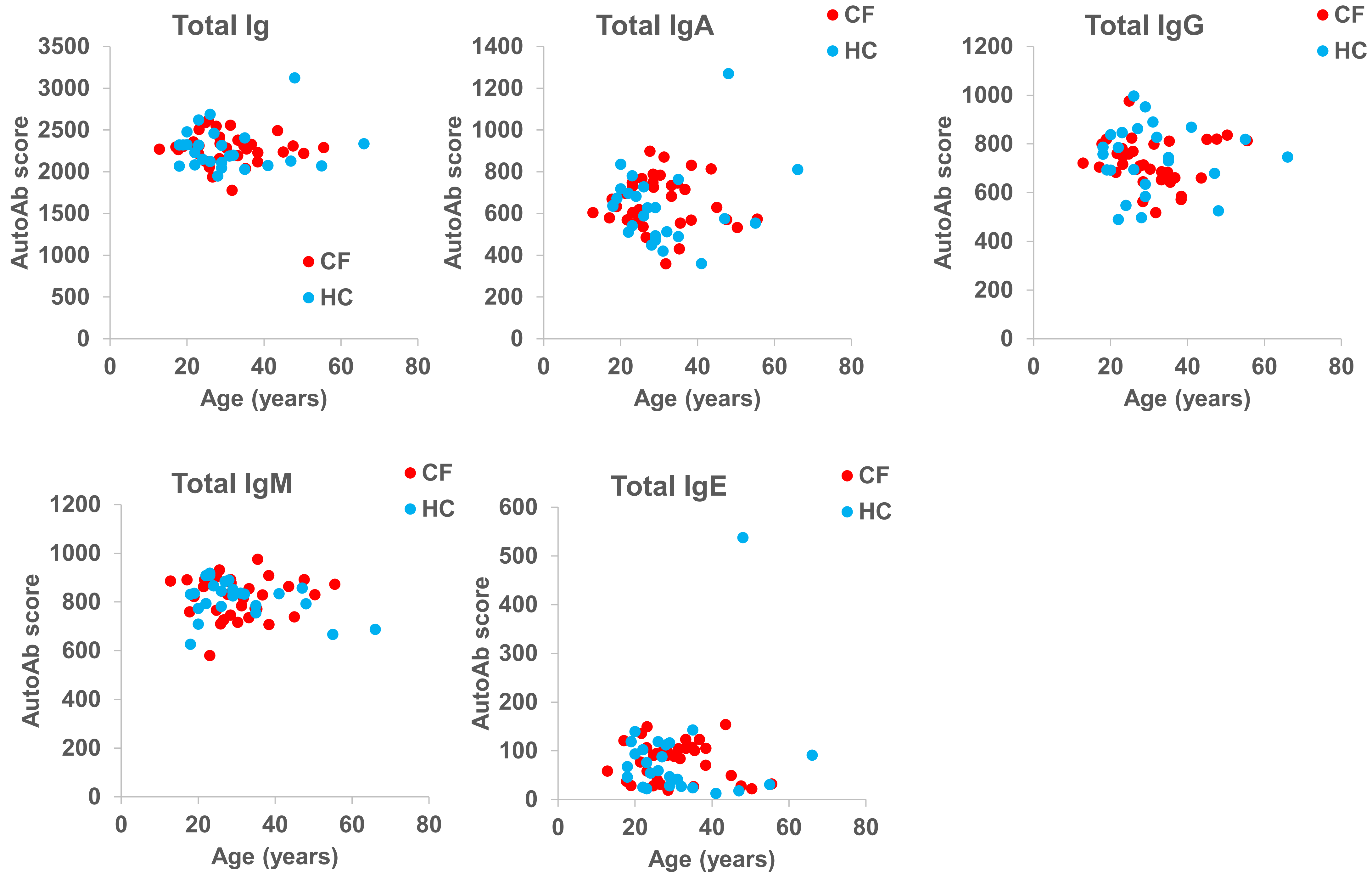

Autoantibody scores were calculated for each Ig class by summarizing their fluorescent values for each autoantigen. The “total Ig” autoantibody score was calculated by summarizing the autoantibody scores of the four classes (IgA+IgG+IgE+IgM). Each dot represents a human subject. CF, PwCF; HC, healthy control.

Suppl. figure 3. Distribution of age and lung function values between PwCF with or without *S. aureus* infection.

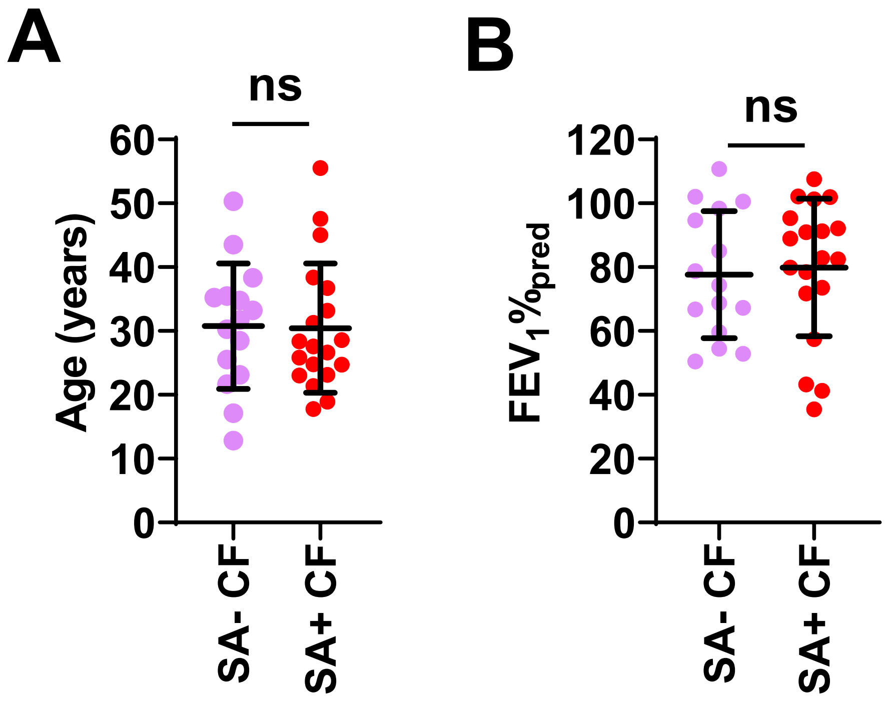

A) Age and **B)** $FEV_1\%_{pred}$ distribution of the SA- and SA+ CF cohorts. Mann-Whitney test. Ns, non-significant; SA, *Staphylococcus aureus*; CF, cystic fibrosis; FEV, forced expiratory volume.

Suppl. figure 4. Anti-PCNA IgM, IgA and IgG autoantibody scores in indicated patient cohorts.

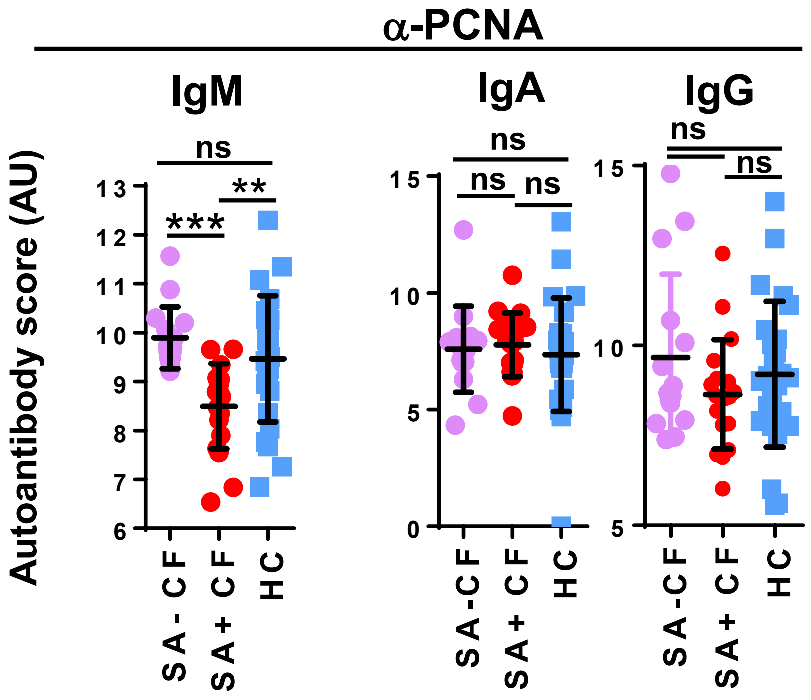

Anti-PCNA IgM, IgA and IgG autoantibody scores are compared between indicated patient cohorts. Each dot represents a human patient. One-way ANOVA and Tukey's multiple comparisons test. **, p<0.01; ***, p<0.001. Ns, non-significant; SA, *Staphylococcus aureus*; CF, cystic fibrosis; HC, healthy control; FEV, forced expiratory volume; AU, arbitrary unit.

Suppl. figure 5. The IgM autoantibody score does not correlate with lung disease in *S. aureus*-negative PwCF

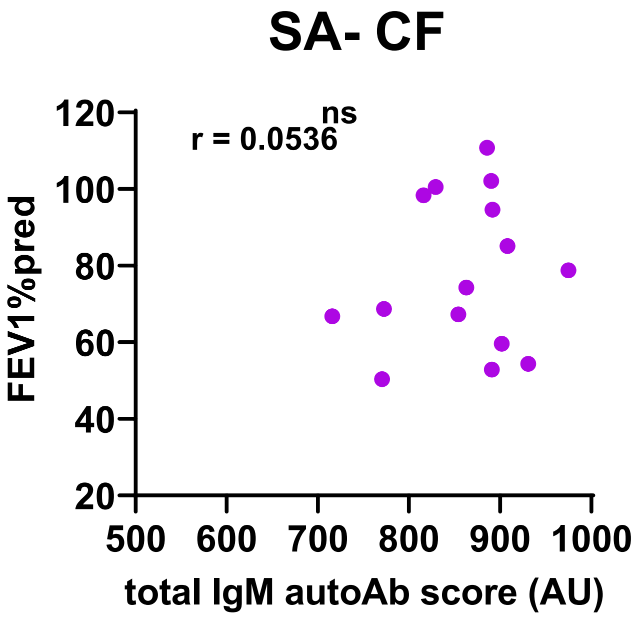

For each of the *S. aureus*-negative PwCF (SA- CF), the total IgM autoantibody score was calculated (X-axis) and correlated with lung function ($FEV_1\%_{pred}$, Y-axis) (r, Spearman correlation coefficient). SA, *Staphylococcus aureus*; FEV, forced expiratory volume; AU, arbitrary unit.

Suppl. Figure 6. Cystic fibrosis-related diabetes is associated with an elevated systemic IgA autoantibody profile

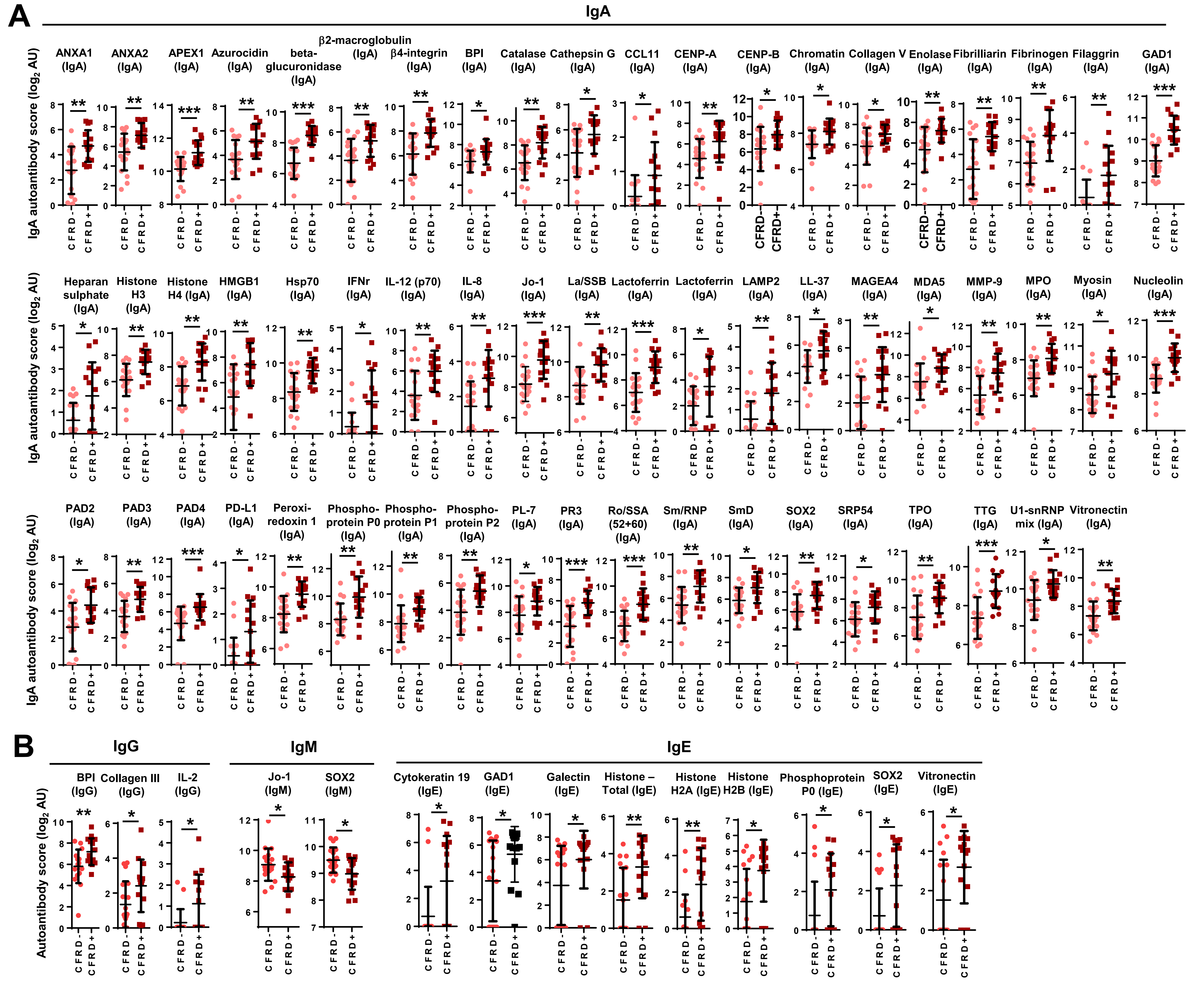

A) IgA autoantibody levels are compared in CFRD+ patients to CFRD- patients for the autoantigens with significant differences between the two groups. Mann-Whitney test.
B) IgG, IgM and IgE autoantibody levels are compared between CFRD+ and CFRD- patients for the autoantigens with significant differences between the two groups. Mann-Whitney test.
*, $p<0.05$; **, $p<0.01$; ***, $p<0.001$. Ns, non-significant; CF, cystic fibrosis; CFRD, CF-related diabetes; HC, healthy control; AU, arbitrary unit.

Supplementary figure 7. Association of *P. aeruginosa* respiratory infection with autoantibodies in CF.

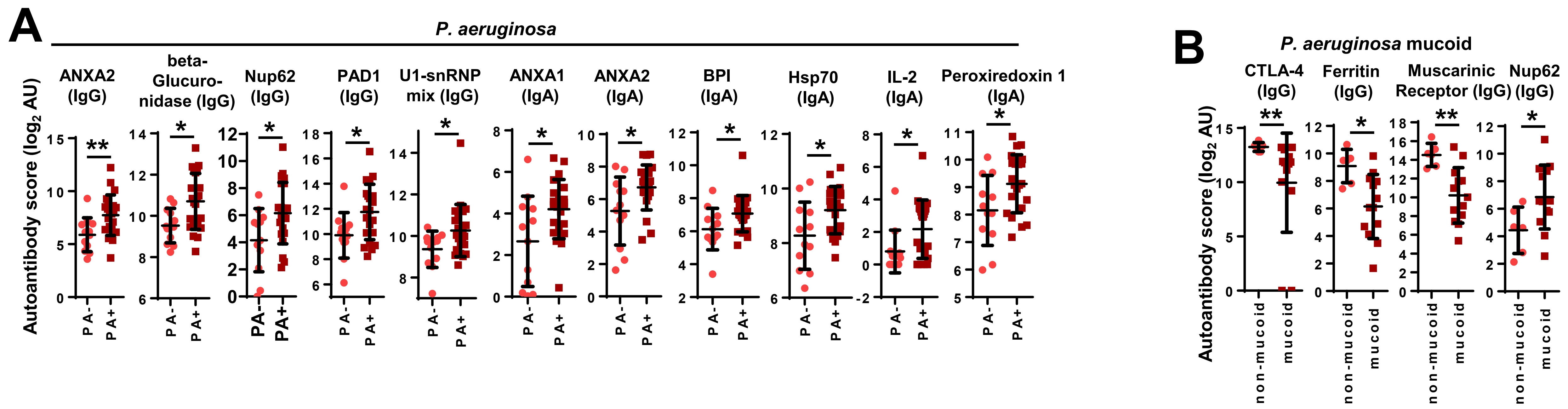

A) Significant differences in autoantibody levels are compared between PwCF with *P. aeruginosa* infection (red) or without *P. aeruginosa* infection (pink) for the indicated autoantigens. **B)** Significant differences in autoantibody levels in *P. aeruginosa*-infected PwCF are compared between patients with mucoid (red) or with non-mucoid (pink) *P. aeruginosa* for the indicated autoantigens. Mann-Whitney test or Spearman correlation coefficient (r). *, $p<0.05$; **, $p<0.01$. CF, cystic fibrosis; AU, arbitrary unit; PA, P. aeruginosa.
